# Prevalence of chronic diseases and morbidity in primary health care in central Greece: An epidemiological study

**DOI:** 10.1186/1472-6963-10-252

**Published:** 2010-08-28

**Authors:** Markos Minas, Nikolaos Koukosias, Elias Zintzaras, Konstantinos Kostikas, Konstantinos I Gourgoulianis

**Affiliations:** 1 Respiratory Medicine Department, University of Thessaly Medical School, Larissa, Greece; 2 Department of Biomathematics, University of Thessaly Medical School, Larissa, Greece

## Abstract

**Background:**

In Greece there is lack of large epidemiological studies regarding morbidity and mortality in primary health care. The aim of the present study was to estimate the prevalence and morbidity of the most common diseases in a large population sample from primary health care.

**Methods:**

Four primary health centres were randomly selected. During one year period, 12 visits were performed in each centre, one per month, in random order and all visitors willing to participate in the study were included. Data on morbidity of each subject were recorded after performing an interview with the participant and checking his medical records, medical history and current medication. Diseases were coded using the international classification of primary care (ICPC) system.

**Results:**

In total 20,299 subjects were recorded. The results revealed significant variations in morbidity between genders and age groups. However, in all age groups, diseases of the circulatory system were most prevalent, followed by endocrine, metabolic, musculoskeletal and respiratory diseases. Osteoporosis was significantly more prevalent in females compared to males, whereas skin and eye disorders were more prevalent in subjects below 65 years old. COPD prevalence was very low compared to worldwide data.

**Conclusions:**

The present study revealed great variations in the prevalence of the diseases between genders and age groups. Our data justify the urgent need for the development of electronic health records that may help in the design of new prevention strategies in primary health care.

## Background

Epidemiological data can be useful in the design of new prevention strategies, especially in primary health care [1]. There are worldwide data available regarding the prevalence, morbidity and mortality of chronic diseases. Data from the World Health Organization (WHO) indicate that the most important causes of death are currently ischemic heart disease and cerebrovascular disease [2].

The development of electronic health records, especially in primary care, may help in the identification of the burden of several diseases in each region. The use of such records may be of great importance for health systems, considering that the North Shore Hospital System in Long Island, New York, announced recently that it will pay an incentive of up to $40,000 to each physician in its network who adopts its electronic health record, paying 50% of the cost to physicians who install an electronic health record that communicates with the hospital and 85% of the cost if the physician also shares de-identified data on the quality of care [3].

On the other hand, the International Classification of Primary Care (ICPC) recently celebrated its 21 years. This coding system is very easy and very useful in primary health care [4-6]. In a recent epidemiological study conducted in Spain, ICPC was used in conjunction with electronic health records, for the estimation of the prevalence of major diseases in the general population [7].

In Greece, the national health system is designed in three levels. Primary health care centres compose the first level which represents the primary health care system. General practitioners are the key component of the primary health care centres. Thus, their role is very important as general practitioners represent the link between public and the national health system. However, the establishment of an integrated primary health care system in Greece is still under development [8].

However, in Greece there is lack of a recording system in primary health care, rendering the design of large epidemiological studies difficult and complex. The aim of this study is to estimate the prevalence and morbidity of ten major chronic diseases in primary health care centres serving a semirural population sample in Thessaly, central Greece. Differences in morbidity between genders as well as between young and elderly were further evaluated.

## Methods

### Study design

Data collection was performed from January to December 2008. Thessaly is a district in central Greece with four prefectures and seventeen primary health care centres with a total population of 740,115 residents [9] that represents approximately 8% of the total population of Greece. All these primary health care centres are part of the national health system and correspond mainly to rural and semirural population. Four primary health centres were randomly selected to be recorded, one from each prefecture. The primary health care centres selected correspond to a population of 126,843 residents (data derived from local authorities). The study group visited one centre per week in order to visit all selected centres in one month. The centre visited in each of the four weeks of each month was chosen in a random order. Overall, twelve visits were performed in each centre.

Study participants were all subjects over 14 years old who visited primary health care centres for any reason and were willing to participate in the study. A structured questionnaire was completed by the study coordinators upon the arrival of each subject. The study questionnaire included questions about demographics, medical history and current medication for chronic diseases. The study coordinators additionally checked the medical records of each patient, in order to record all chronic diseases in detail.

The chronic diseases of each subject were identified and coded using the ICPC system [4]. Each disease recorded was included in the respective organic system according to ICPC codes (Table 1). The ten most common and burdensome chronic diseases, based on the main causes of death and disease burden in the United States and the most common diagnoses in primary care, were recorded separately [10]. The study was approved by the Ethics committee of the University Hospital of Larissa and all subjects provided informed consent.

**Table 1:** ICPC codes for selected diseases and organic systems

A. General	
B. Blood, lymphatics	

C. Digestive	

F. Eye	

H. Ear	

K. Circulatory	

Elevated blood pressure	K85, K86, K87

Coronary heart disease	K74, K75

L. Musculoskeletal	

Osteoarthritis	L83, L84, L89, L90, L91

Osteoporosis	L95

N. Neurological	

P. Psychological	

Depression	P03, P76, P78

Anxiety disorders	P01, P74, P75, P79, P82

R. Respiratory	

Asthma	R96

COPD	R79, R95

T. Endocrine, metabolic and nutritional	

Lipid disorder	T93

Diabetes	T89, T90

U. Urology	

X. Genital Female	

Y. Genital male	

### Statistical analysis

Demographic data are presented as median (interquartile range) whereas categorical variables are presented as percentages. Normality of data was estimated with the use of D'Agostino-Pearson normality test. Comparison between medians was performed using Mann-Whitney U test for skewed data. The prevalence was estimated taking into account the cluster design and based on the cluster sample total [11,12]. The analysis was performed using the proc survey means of SAS v.90 and GraphPad Prism v.5.0. The prevalence of each disease is presented per 10,000 of population.

## Results

Demographic data are presented in Table 2. In total, 20,299 subjects were willing to participate in the study (56.4% female). Participants were distributed according to the gender and age group (< 65 years, ≥65 years). According to Table 2, the majority of participants were elderly women. In the age group < 65 years, males were younger than females (48 vs. 51 years old), whereas in the age group ≥65 years old males were older than females (74 vs. 73 years old).

**Table 2:** Demographic data of the study participants

	Male			Female			Total
	**< 65 years**	**≥65 years**	**Total**	**< 65 years**	**≥65 years**	**Total**	

N	3703	5155	8858	4720	6721	11441	20299

Age	48 (35-58)*	74 (70-79)^	68 (52-75)	51 (36-60)*	73 (69-78)^	68 (55-74)	68 (54-75)

Disease number							

0	9 (0.24%)	5 (0.10%)	14 (0.16%)	32 (0.68%)	4 (0.06%)	36 (0.31%)	50

1	3341 (90.22%)	3732 (72.40%)	7073 (79.85%)	4001 (84.77%)	4522 (67.28%)	8523 (74.50%)	15596

2	288 (7.78%)	1087 (21.09%)	1375 (15.52%)	544 (11.52%)	1581 (23.53%)	2125 (18.57%)	3500

3	62 (1.67%)	273 (5.30%)	335 (3.78%)	128 (2.71%)	512 (7.62%)	640 (5.59%)	975

4	3 (0.09%)	54 (1.05%)	57 (0.64%)	14 (0.30%)	91 (1.35%)	105 (0.92%)	162

5	0 (0%)	4 (0.06%)	4 (0.05%)	1 (0.02%)	10 (0.15%)	11 (0.1%)	15

6	0 (0%)	0 (0%)	0 (0%)	0 (0%)	1 (0.01%)	1 (0.01%)	1

*,^ P < 0.0001

**Figure 1:**
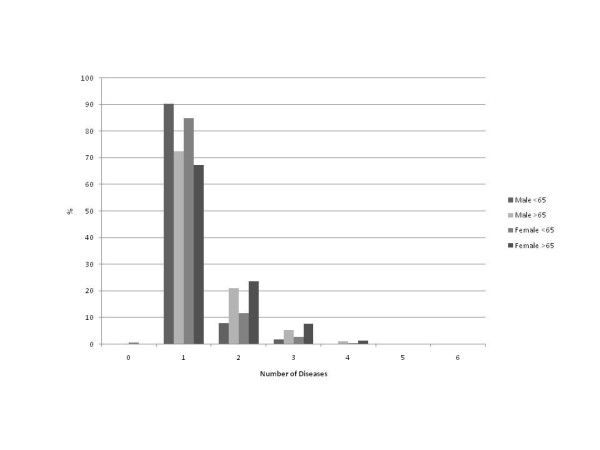
**Distribution of disease numbers between genders and age groups**.

Table 2 and Figure 1 present the distribution of participants according to their number of diseases. In all age groups the majority of participants suffered from a single disease, however with several variations. For example, a significant proportion of people ≥65 years old had two or more diseases. Moreover, women had more commonly 2 or more diseases in both age groups.

The estimated prevalence of each system or disease, expressed per 10000 people, is shown in Table 3. The most prevalent diseases overall were circulatory disorders, including arterial hypertension and coronary heart disease. Other diseases with high prevalence were endocrine, metabolic (especially lipid disorders), musculoskeletal and respiratory diseases. Interestingly, osteoarthritis and asthma, two chronic diseases classified in the two latter organic systems, presented low prevalence. It should be mentioned also that the prevalence of chronic obstructive pulmonary disease (COPD) was considerably low compared to other diseases.

**Table 3:** Estimated prevalence of the recorded diseases

	N	Estimated prevalence (per 10,000 residents)	95% CI
A. General	1099	552.0	(471.0 - 633.0)

B. Blood, lymphatics	486	240.6	(175.0 - 306.2)

C. Digestive	1688	844.6	(673.2 - 1016.1)

F. Eye	451	222.8	(167.1 - 278.6)

H. Ear	207	104.8	(45.5 - 164.1)

K. Circulatory	7026	3409.2	(2959.4 - 3860.3)

Elevated blood pressure	3719	1813.2	(1505.2 - 2121.1)

Coronary heart disease	2321	1107.2	(710.2 - 1504.2)

L. Musculoskeletal	3150	1551.4	(1448.1 - 1654.7)

Osteoarthritis	684	340.0	(249.6 - 430.4)

Osteoporosis	1047	500.7	(403.3 - 598.0)

N. Neurological	975	489.0	(420.4 - 557.6)

P. Psychological	1968	949.9	(669.8 - 1230.0)

Depression	580	269.1	(79.9 - 458.2)

Anxiety disorders	376	191.4	(132.4 - 250.4)

R. Respiratory	3122	1548.3	(1353.7 - 1742.9)

Asthma	103	53.8	(34.2 - 73.4)

COPD	706	349.6	(277.7 - 421.6)

S. Skin	927	452.7	(381.9 - 523.6)

T. Endocrine, metabolic and nutritional	4058	1945.1	(1641.7 - 2248.5)

Lipid disorder	2026	960.4	(741.4 - 1179.4)

Diabetes	1472	714.6	(646.9 - 782.4)

U. Urology	545	268.8	(193.6 - 344.0)

X. Genital Female	242	120.7	(99.4 - 142.1)

Y. Genital male	302	147.5	(114.7 - 180.3)

CI: confidence intervals

### Differences between genders

The comparison of the estimated prevalence of the diseases revealed significant differences between the two genders, as presented in Table 4. The most prominent difference is the higher prevalence of the diseases of musculoskeletal system in women, being more than 10-fold higher for osteoporosis. Another important difference is the fact that psychological diseases, especially depression, were more prevalent in females. Circulatory diseases were slightly more prevalent in females, with the exception of coronary heart disease which was slightly more common in males. Another interesting finding is the higher prevalence of COPD compared to asthma in females.

**Table 4:** Estimated prevalence for each disease according to gender and age group

	Male						Female						Total
	**< 65 years**		**≥65 years**		**Total**		**< 65 years**		**≥65 years**		**Total**		

	**N**	**Prevalence (95% CI)**	**N**	**Prevalence (95% CI)**	**N**	**Prevalence (95% CI)**	**N**	**Prevalence (95% CI)**	**N**	**Prevalence (95% CI)**	**N**	**Prevalence (95% CI)**	

A.General	460	1262.5 (1107.5 - 1417.5)	183	358.2 (250.5 - 465.9)	643	738.0 (617.1 - 859.0)	278	591.8 (522.2 - 661.5)	178	275.4 (214.6 - 336.2)	456	407.0 (353.9 - 460.0)	1099

B.Blood, lymphatics	31	85.8 (60.4 - 111.1)	119	246.7 (143.2 - 350.3)	150	173.1 (133.0 - 213.3)	124	256.4 (172.9 - 339.8)	212	322.2 (214.9 - 429.5)	336	292.9 (199.8 - 386.0)	486

C.Digestive	341	937.8 (804.4 - 1071.2)	431	822.9 (585.0 - 1060.7)	772	876.7 (701.1 - 1052.3)	398	859.1 (707.3 - 1010.8)	518	788.5 (582.0 - 995.0)	916	820.4 (642.7 - 998.2)	1688

F.Eye	89	238.8 (157.3 - 320.3)	117	231.1 (178.2 - 284.0)	206	233.1 (173.5 - 292.7)	56	119.0 (114.8 - 123.1)	189	282.0 (188.8 - 375.3)	245	215.2 (155.1 - 275.3)	451

H.Ear	58	155.5 (90.2 - 220.7)	31	66.6 (23.9 - 109.4)	89	105.0 (50.9 - 159.1)	78	158.4 (56.9 - 260.0)	40	61.9 (12.8 - 111.0)	118	104.7 (31.3 - 178.2)	207

K.Circulatory	715	1940.0 (1730.1 - 2149.9)	2445	4700.9 (4485.4 - 4916.4)	3160	3532.6 (3186.1 - 3879.1)	890	1892.2 (1494.8 - 2289.6)	2976	4344.2 (3959.3 - 4729.2)	3866	3311.7 (2775.2 - 3848.1)	7026

Elevated blood pressure	389	1062.6 (868.6 - 1256.5)	1096	2105.5 (1743.4 - 2467.5)	1485	1665.3 (1362.0 - 1968.5)	589	1251.2 (1006.4 - 1496.0)	1645	2422.7 (2087.1 - 2758.2)	2234	1926.8 (1595.3 - 2258.4)	3719

Coronary heart disease	225	608.7 (369.6 - 847.7)	959	1817.3 (1351.9 - 2282.6)	1184	1308.9 (890.7 - 1727.2)	179	380.8 (148.0 - 613.6)	958	1354.4 (946.4 - 1762.5)	1137	949.3 (562.4 - 1336.2)	2321

L.Musculoskeletal	416	1122.3 (1047.1 - 1197.5)	457	886.9 (826.0 - 947.8)	873	988.0 (926.4 - 1049.6)	795	1696.8 (1491.5 - 1902.0)	1482	2216.7 (2017.8 - 2415.6)	2277	1990.4 (1795.6 - 2185.3)	3150

Osteoarthritis	60	163.3 (131.8 - 194.7)	121	234.5 (171.9 - 297.0)	181	203.1 (158.4 - 247.7)	145	315.4 (238.3 - 392.5)	358	537.7 (366.9 - 708.5)	503	447.2 (316.8 - 577.6)	684

Osteoporosis	11	27.6 (0 - 55.7)	37	68.8 (36.3 - 101.3)	48	51.0 (20.3 - 81.7)	300	629.3 (499.4 - 759.3)	699	1014.4 (911.7 - 1117.1)	999	850.3 (712.8 - 987.8)	1047

N.Neurological	122	328.9 (284.5 - 373.2)	278	545.2 (426.0 - 664.5)	400	456.1 (393.7 - 518.6)	216	466.6 (391.3 - 542.0)	359	550.6 (462.5 - 638.7)	575	515.4 (434.5 - 596.4)	975

P.Phychological	321	873.9 (696.7 - 1051.1)	396	733.3 (472.5 - 994.1)	717	792.3 (571.6 - 1031.1)	498	1074.1 (726.5 - 1241.7)	753	1078.6 (758.1 - 1399.0)	1251	1075.5 (752.5 - 1398.5)	1968

Depression	42	111.3 (21.4 - 201.3)	115	201.2 (39.9 - 362.6)	157	165.0 (30.3 - 299.7)	141	305.2 (104.7 - 505.7)	282	379.4 (129.9 - 628.9)	423	349.9 (117.5 - 582.2)	580

Anxiety disorders	77	212.1 (125.1 - 299.0)	68	128.5 (77.6 - 179.3)	145	168.5 (120.5 - 216.5)	115	248.5 (162.9 - 334.2)	116	179.7 (102.9 - 256.5)	231	209.1 (137.1 - 281.2)	376

R.Respiratory	717	1887.6 (1529.3 - 2245.9)	780	1548.0 (1316.7 - 1779.4)	1497	1692.3 (1503.0 - 1881.7)	937	1969.7 (1646.7 - 2292.6)	688	1029.0 (996.3 - 1061.6)	1625	1436.4 (1234.6 - 1638.3)	3122

Asthma	15	40.6 (20.5 - 60.8)	14	29.6 (4.0 - 55.2)	29	33.9 (16.2 - 51.7)	40	87.4 (58.7 - 116.1)	34	57.4 (23.9 - 90.9)	74	69.4 (41.1 - 97.6)	103

COPD	71	188.3 (144.1 - 232.5)	326	640.6 (545.9 - 735.4)	397	451.9 (392.7 - 511.0)	79	172.7 (120.8 - 224.7)	230	332.3 (212.0 - 452.5)	309	269.4 (184.3 - 354.5)	706

S.Skin	286	779.3 (671.5 - 887.2)	158	294.2 (208.2 - 380.2)	444	497.1 (429.4 - 564.7)	290	612.7 (526.7 - 698.7)	193	273.2 (178.5 - 367.8)	483	418.0 (330.2 - 505.7)	927

T.Endocrine, metabolic and nutitional	413	1096.5 (941.4 - 1251.5)	1183	2268.6 (2018.8 - 2518.3)	1596	1770.2 (1556.5 - 1983.8)	712	1476.0 (1124.8 - 1827.3)	1750	2525.0 (2214.5 - 2834.5)	2462	2080.4 (1696.6 - 2464.2)	4058

Lipid disorder	233	620.6 (520.3 - 720.9)	572	1092.5 (1005.7 - 1179.3)	805	892.9 (791.4 - 994.4)	358	723.9 (401.0 - 1046.8)	863	1223.8 (954.8 - 1492.8)	1221	1010.9 (698.7 - 1323.0)	2026

Diabetes	137	362.6 (282.5 - 442.6)	474	925.3 (774.5 - 1076.0)	611	680.9 (587.1 - 774.6)	208	435.1 (354.1 - 516.1)	653	698.2 (938.8 - 997.6)	861	741.7 (678.9 - 804.4)	1472

U.Urology	96	255.4 (165.0 - 345.8)	131	262.7 (152.0 - 373.4)	227	259.0 (158.2 - 359.8)	140	290.9 (220.6 - 361.1)	178	264.0 (218.4 - 309.7)	318	276.2 (220.0 - 332.5)	545

X.Genital Female	-	-	-	-	-	-	122	261.2 (196.0 - 326.4)	124	179.8 (153.3 - 206.3)	246	217.4 (180.6 - 254.3)	246

Y.Genital male	50	140.3 (96.8 - 183.7)	252	470.4 (373.1 - 567.8)	302	333.9 (254.0 - 413.8)	-	-	-	-	-		302

CI: Confidence interval. Estimated prevalence corresponds to each gender and age group and is expressed per 10000 residents

### Differences between age groups

Significant differences in the prevalence of the diseases between the young and the elderly were observed (Table 4). Of great importance were the differences in the general diseases, the diseases of the skin and the respiratory diseases, which were more prevalent in younger patients. In contrast, circulatory disorders were more prevalent in the elderly. Surprisingly, psychological disorders had similar overall prevalence in both genders and in both age groups, whereas anxiety disorders were more prevalent in the younger.

The differences between genders in each age group were not very prominent. In younger patients, circulatory diseases had similar prevalence between males and females, whereas coronary heart disease was significantly more prevalent in males. The same pattern was observed in the elderly, but with a more blunted difference. Musculoskeletal diseases were more prevalent in females in both age groups; however, the difference in the elderly was more prominent. Interestingly, the prevalence of COPD was similar in the two genders of the younger group, whereas it was more prevalent in elderly males compared to females.

## Discussion

The results of the present study indicate that circulatory and metabolic diseases represent the most important causes of morbidity in the general population visiting primary health care centres, followed by endocrine, metabolic, musculoskeletal and respiratory diseases. Significant variations in the prevalence of diseases between genders and age groups were observed. Our results support the fact that in Greece there is an unmet need for the development of a recording system in primary health care, in order the pattern of diseases in specific areas and age groups to be identified and effective strategies for the prevention and management of individual patients to be implemented.

The primary health care facilities of the National Health System in Greece are located mainly in rural areas. However, recently the first urban primary health centre was introduced in the Greek health care system, and Mariolis et al. showed that there are variations between urban and rural primary health centres [13]. The primary health centres selected for this study correspond to a rural and semirural population, since there are no urban primary health care centres in Thessaly.

The distribution of the diseases estimated in this study is similar with worldwide data. Data from WHO indicate that cardiovascular disease and diabetes mellitus are important causes for morbidity and mortality worldwide [2] and cardiovascular diseases are the leading cause of death worldwide, especially in women. In low and middle income countries of Europe in the age group of 15-59 years old, two thirds of all deaths are associated with cardiovascular diseases, cancer and other non-communicable diseases [2]. It is estimated that the global cardiovascular deaths will increase to 11.8 million in 2030 [2]. Data from the present study are in accordance with these data, since the results of this study indicate that the diseases of the circulatory system are the most prevalent in all age groups, both in males and females.

Another important group of diseases of high prevalence in the Greek population are neuropsychiatric disorders. Mental disorders are an important source of lost years of healthy life among women 15 to 44 years old [2]. Moreover, neuropsychiatric disorders have been observed also in the 16.5% of a representative sample of South African adults [14]. Our data indicate that the prevalence of depression is higher in women compared to men, in accordance with previous studies.

Recent data from a Spanish population have shown that the prevalence of chronic health problems in general practice was higher in women and increased with age [7], a finding similar to the present study. Arterial hypertension was the most prevalent disease in the Spanish study [7], and this was also the case in two other studies from Italy and Sweden [15,16]. It is important to be noticed that these studies were performed in primary care, since data from secondary and tertiary care are additionally needed for the correct estimation of the disease prevalence [16]. A previous study in a random nationwide sample of adult Greek population has also indicated that self-reported arterial hypertension represents a significant public health problem [17].

One important difference between the present and the Spanish study is that COPD is more prevalent in Spain compared to our sample of Greek population [7]. The prevalence of COPD depends on the smoking pattern of each country [18], despite the fact that there is an increasing number of studies showing high rates of COPD in non-smokers [19]. There is a high prevalence of smoking in urban areas of Greece [20], which is lower in rural areas [21]. The fact that smoking rates are lower in the rural areas recorded in the present study, in combination with the underdiagnosis of COPD in Greece [22] may account for this discrepancy.

Several regions worldwide present a totally different pattern of diseases compared to our findings. For example, data from African studies indicate that the major causes of death in sub Saharan regions were communicable, maternal and perinatal conditions [23]. Despite the fact that in these regions the pattern of morbidity and mortality is entirely different, a rise in non communicable diseases has been recently observed [14].

Epidemiologic data depend on the presence of recording systems. Several studies have emphasized the usefulness of electronic health records in physicians' practice. The development of a new pan-Canadian network for primary health care has been recently announced [24]. However, there is not adequate adoption of these records, even in US hospitals [25], and the proportion of physicians that use them remains very low, even in primary care settings [3]. The absence of epidemiologic data on many chronic diseases in Greece renders the conduction of large epidemiologic studies in general population imperative, in order to design new strategies in primary care.

Despite the large number of participants, the present study presents several limitations. Firstly, due to geographic reasons, our sample may present significant differences compared to the general population. Data from Eurostat indicate that in Greece the proportion of the population aged > 65 years old is 18.6% [26], whereas in this study patients ≥65 years old represent over 50% of our sample. On the other hand the selected primary health care centres correspond to rural and semirural areas, with possible differences in the distribution of the population compared to urban areas. However, it should be mentioned that our study population represents approximately 16% of the population of the area that corresponds to the health care centres involved. Another possible bias is that the participation in the study was voluntary after invitation. Although there are no data on the morbidity of the subjects that were not willing to participate, the large number of participants, as well as the large number and the random order of visits in each health centre may minimize this factor. The additional evaluation of the patients' health records may have further diminished the inaccurate recording of chronic diseases.

## Conclusions

In conclusion, the burden of chronic diseases in Greece seems to be similar with the burden observed in Europe, and especially in the middle and high income countries. Arterial hypertension, diabetes mellitus, diseases of the circulatory system and depression seem to be the most prevalent diseases. The present study revealed great variations in the prevalence of the diseases between genders and age groups. Our data justify the urgent need for the development of electronic health records that may help in the design of new prevention strategies in primary health care.

## Abbreviations

COPD: Chronic Obstructive Pulmonary Disease; ICPC: International Classification of Primary Care; WHO: World Health Organization.

## Competing interests

The authors declare that they have no competing interests.

## Authors' contributions

MM performed analysis of data and drafted the manuscript; NK contributed in the study design and acquisition of data; EZ performed the statistical analysis; KK contributed in the conception and design of the study and revised the manuscript; KIG was involved in the study conception and gave the final approval for the manuscript. All authors have read and approved the final version of the manuscript.

## Pre-publication history

The pre-publication history for this paper can be accessed here:

http://www.biomedcentral.com/1472-6963/10/252/prepub
